# Supplementary material for: Eudermic Properties and Chemical–Physical Characterization of Honeys of Different Botanical Origin
Source: Nutrients. 2024 Oct 26;16(21):3647. doi: 10.3390/nu16213647 (PMC11547790; doi:10.3390/nu16213647)
Supplement: Supplementary file 1 [file nutrients-16-03647-s001.zip › nutrients-3232331-supplementary.pdf]

SUPPLEMENTARY

Table S1. Chromatographic separation conditions for the determination of amino acids with HPLC.

Column	Mobile phase		Temperature (°C)	Volume injection (µl)	excitation wave length (nm)	emission wave length (nm)	Gradient (flow rate 1.0 ml/min)
Supelco Ascentis C18 column, 250 mm×4.6 mm×5 µm.	A:10.42 g/l sodium acetate in water with 0.19 % TEA, titrated to pH 5 with phosphoric acid (0.4%	B: acetonitrile /water 60/40 v/v	40	10	250	395	Time 0 100% A; 0.84 min 98% A (curve 6); 25 min 93% A (curve 6); 31.7 min 90% A (curve 6); 53.4 min 67% A; 55.1 min 67% A (curve 6); 61.8 min 75% A (curve 6); 63.5 min 100% A (curve 6); 70 min 100% A

Table S2 Data on the variance explained by each principal component, the cumulative proportions and their standard deviations.

```
Importance of components:
                          PC1    PC2    PC3     PC4     PC5     PC6    PC7
Standard deviation     3.1602 2.7476 1.9005 1.30465 1.18349 1.10625 0.7247
Proportion of Variance 0.3841 0.2904 0.1389 0.06547 0.05387 0.04707 0.0202
Cumulative Proportion  0.3841 0.6745 0.8134 0.87886 0.93273 0.97980 1.0000
                             PC8
Standard deviation     7.227e-16
Proportion of Variance 0.000e+00
Cumulative Proportion  1.000e+00
```
